# Corrigendum to “Does Comorbidity Increase the Risk of Dengue Hemorrhagic Fever and Dengue Shock Syndrome?”

**DOI:** 10.1155/2017/2725850

**Published:** 2017-11-22

**Authors:** Shahid Mahmood, Saadia Hafeez, Hibbah Nabeel, Urooj Zahra, Hammad Nazeer

**Affiliations:** ^1^Department of Community Medicine, Gujranwala Medical College, Gujranwala, Pakistan; ^2^Fatima Jinnah Medical College, Lahore, Pakistan; ^3^Department of Infectious Diseases, Shaukat Khanum Memorial Hospital, Lahore, Pakistan

In the article titled “Does Comorbidity Increase the Risk of Dengue Hemorrhagic Fever and Dengue Shock Syndrome?” [1], the name of the third author was given incorrectly as Hiba Nabeel. The author's name should have been written as Hibbah Nabeel. The revised authors' list is shown above.

## References

[1] Mahmood S., Hafeez S., Nabeel H., Zahra U., Nazeer H. (2013). Does comorbidity increase the risk of dengue hemorrhagic fever and dengue shock syndrome?. *ISRN Tropical Medicine*.

